# Endogenous CD5L controls the metabolic and inflammatory state of human macrophages

**DOI:** 10.3389/fimmu.2025.1677948

**Published:** 2026-01-22

**Authors:** Kashif Rasheed, Neda Nejati Moharrami, Erlend Bjørkøy Tande, Terje Espevik, Maria Yurchenko, Victor Boyartchuk

**Affiliations:** 1 Centre of Molecular Inflammation Research (CEMIR), Department of Clinical Research and Molecular Medicine (IKOM), Faculty of Medicine and Health Sciences (MH), Norwegian University of Science and Technology (NTNU), Trondheim, Norway; 2 Clinic of Surgery, St Olav Hospital HF, Trondheim, Norway

**Keywords:** CD5L, cellular lipidome, inflammation, monocyte, RORA

## Abstract

**Introduction:**

CD5L is a scavenger receptor-like molecule that mediates diverse physiologic processes, including cell survival, atherogenesis, inflammation, and lipid metabolism. Even though CD5L is an abundant circulatory protein, it has recently become apparent that its expression can alter inflammatory signaling in a cell-autonomous fashion. To date, the effect of endogenous *CD5L* expression in human macrophages remains largely unexplored. Our work addressed this question by analyzing the impact of *CD5L* gene disruption on the inflammatory state of the THP-1 human monocytic cell line.

**Results:**

In macrophage-like *CD5L*-knockout cells, we observed a dramatic decrease in the basal expression of a subset of NF-κB-regulated genes when compared to control cell lines. These differences persisted after stimulation with lipopolysaccharide (LPS), even though the magnitude of induction was similar in both mutant and control cells. Consistent with the lipid remodeling function attributed to CD5L, we found significant changes in the makeup of the intracellular lipid pool. However, we did not detect significant changes in the activity of fatty acid synthase, which has been suggested to mediate CD5L lipidome remodeling function. Furthermore, we explored how CD5L function impacts undifferentiated monocytes. We found that in undifferentiated, unstimulated monocytes deleted for CD5L, several dysregulated transcripts code for genes involved in cell-to-cell interactions and in the progression of atherosclerosis. Most importantly, we found that CD5L deletion upregulates the expression of CD52, a novel anti-inflammatory switch.

**Discussion:**

Overall, our findings further support the multifunctional nature of CD5L and, for the first time, suggest its involvement in monocyte localization to sites of future lesions.

## Introduction

Innate immune signaling has numerous ways to sense changes in cellular metabolism to ensure an appropriate response to threatening agents. For instance, cholesterol metabolites act as ligands for nuclear receptor transcription factors, such as liver X receptors (LXRs), which primarily control cholesterol homeostasis (1). In addition, LXRs link cholesterol metabolism to inflammatory responses. In the presence of 22(*R*)-hydroxycholesterol, LXRs can inhibit LPS induction of IL-6, while an increase in 25-hydroxycholesterol levels results in LXR-mediated induction of IL-6 in the context of herpes simplex-1 virus infection (2, 3). LXRs have a wide range of targets, some of which serve as additional connection points between immune and metabolic signaling. One of such genes is *CD5L*, which encodes a scavenger receptor-like molecule that has recently been recognized to play an important role in the control of inflammatory signaling through changes in cellular metabolism (4–6).

CD5L, also known as apoptosis inhibitor of macrophages (AIM), Api6, and Spα, is a member of the scavenger receptor cysteine-rich (SRCR) superfamily (7). CD5L is a secreted protein, which in mice is mostly expressed in macrophages (8). The cellular source of human protein has not been defined yet, and, in contrast to mice, human macrophages produce relatively low levels of CD5L. Even though the tissue distribution of *CD5L* mRNA is similar in human and mouse, the abundance of the transcript differs in the two species (9). In circulation, CD5L is mostly associated with IgMs, which allows it to escape renal excretion and maintain its high serum concentration (~60 μg/mL) (10–12). CD36 scavenger receptor has been suggested as the CD5L receptor in mice (13). However, non-CD36-expressing cells, such as thymocytes and natural killer T (NKT) cells, can respond to CD5L, suggesting the existence of alternative receptors (14).

CD5L is a multifunctional molecule that plays a role in various systemic and cellular processes, including cell survival, atherogenesis, and inflammation. Murine *Cd5l* was first discovered as an anti-apoptotic gene in thymocytes, and it was initially named as AIM (14). Subsequently, the anti-apoptotic role of CD5L was further described in murine T and NKT cells and human macrophages (15, 16). Another piece of evidence for the pro-survival role of CD5L was provided by Zou et al., demonstrating CD5L’s control of pyroptosis through the inhibition of Caspase-1 activation (17).

Multiple activities of CD5L underlie its substantial impact on the pathogenesis of systemic diseases, such as atherosclerosis. CD5L has been shown to promote atherogenesis by driving distinct steps in foam cell formation. First, CD5L, as a scavenger receptor-like molecule, binds oxidized-LDL and facilitates its uptake through CD36 (16). Second, the anti-apoptotic function of CD5L inhibits the death of lipid-laden macrophages within the artery wall and thus seeds the development of atherosclerotic plaque (18). Both these functions promote foam cell formation and persistence.

CD5L has a well-documented role in the control of inflammatory signaling, with both pro- and anti-inflammatory functions assigned to this molecule. Sanjurjo et al. showed that CD5L inhibits *TNF* and *IL-1β* expression in THP-1 cells (19). In a murine model, Wang et al. discovered that *Cd5l* endogenous expression levels alter the lipid content of Th17 cells and thus determine their inflammatory state (6). However, several reports have documented the pro-inflammatory role of CD5L. Nishikido et al. showed that the deletion of *Cd5l* attenuates the inflammatory response in a murine model of acute myocardial infarction (20). Furthermore, an increase in serum CD5L has been demonstrated to be critical for macrophage recruitment and inflammation in adipocytes (21).

One of the key roles of CD5L is the regulation of lipid homeostasis. In adipocytes, CD5L induces lipolysis by inhibiting fatty acid synthase (FASN) enzyme. FASN is responsible for the synthesis of fatty acids, and thus, the inhibition of its activity leads to a decrease in fatty acid content in adipocytes (13). As a result, this inhibition leads to increased efflux of free fatty acids from adipocytes and subsequent inflammation (21). Furthermore, Wang et al. demonstrated that the inflammatory state of murine Th17 cells reflects the presence or absence of endogenous *Cd5l* expression. In these cells, *Cd5l* expression drives changes in intracellular lipid content and thus determines the availability of RORγt nuclear receptor ligands. Of note, despite abundant circulating Cd5l in the serum, it is intracellular Cd5l that appears to define this lipidome remodeling function (6). RORγt, which senses the lipid remodeling function of CD5L in Th17 cells, is a member of the RAR-related orphan receptor (ROR) family of nuclear receptor transcription factors. RORs, just like LXRs, tie together metabolic and inflammatory signaling pathways. In mammals, there are three major isotypes of RORs: α, β, and γ (22–25). Human macrophages produce mostly RORα, which is encoded by the *RORA* gene (26). RORα, also known as NR1F1, is widely expressed in tissues including the brain, thymus, heart, vessels, and liver. It is a multifunctional transcription factor that has been shown to play important roles in cerebellar development, osteogenesis, atherogenesis, and inflammation (27–34). In murine immune cells, RORα has been shown to play an anti-inflammatory role by inducing IκBα, a negative regulator for the NF-κB signaling pathway (29, 35). Furthermore, our earlier work has established that RORα plays an anti-inflammatory role in human macrophages (36).

Accumulated data documenting the involvement of CD5L in inflammatory signaling underscore the importance of understanding the scope, direction, and magnitude of its regulatory effects in relevant cell types. In our study, we hypothesized that the inflammatory state in macrophages is controlled by CD5L, which induces changes in the lipidome that can be sensed by RORα, similarly to how RORγt acts in murine Th17 cells. Here, we used the deletion of *CD5L* as a tool to characterize its role in human macrophage-like cells.

## Materials and methods

### Reagents

Penicillin-Streptomycin (P0781), phorbol 12-myristate 13-acetate (PMA) (P8139), βME (M3148), Hank’s balanced salt solution (H9269), Dulbecco's Phosphate Buffered Saline (DPBS) (D8537), polybrene (107689), 4-(2-hydroxyethyl)-1-piperazineethanesulfonic acid (HEPES) (H3375), malonyl coenzyme-A lithium salt (M4263), acetyl coenzyme-A lithium salt (A2181), β-nicotinamide adenine dinucleotide 2′-phosphate reduced tetrasodium salt hydrate (N1630), and potassium phosphate (795488) were purchased from Sigma-Aldrich/ Merck Darmstadt, Germany. Dulbecco's Modified Eagle Medium (DMEM) (BE12-604F), Trypsin/EDTA (BE17-161E, Lonza, Bazel, Switzerland), Fetal Bovine Serum (10270-106, GIBCO, Thermo Fisher Scientific, Waltham, MA, USA), and LPS-EK Ultrapure from the *Escherichia coli* K12 strain (tlrl-peklps) were from InvivoGen, San Diego, CA, USA. Opti-MEM™ I Reduced Serum Medium (11058021) was from Thermo Fisher Scientific, and puromycin (540222) and sucrose (57-50-1) were from VWR, Randor, PA, USA. Dithiothreitol (DTT) (A3668) was from AppliChem, Darmstadt, Germany, cOmplete EDTA-free protease inhibitor cocktail tablets (05056489001) were from Roche Diagnostics GmbH, Mannheim, Germany, and protein assay dye reagent concentrate (5000006) was from Bio-Rad., Hercules, CA, USA. *Bsm*BI restriction enzyme (R0580), T4 DNA ligase (M0202), and T4 DNA ligase reaction buffer (B0202) were from New England Biolabs, Ipswich, MA, USA. The PureYield Plasmid Miniprep System (A1222) was from Promega.

### Cells

THP-1 human monocytic cell line (ATCC, TIB-202) was maintained in RPMI 1640 (A10491-01, GIBCO) medium supplemented with 10% FBS, 100 U/mL penicillin, 0.1 mg/mL streptomycin, and 0.05 mM βME at 37°C in a humidified atmosphere with 5% CO_2_. THP-1 monocytes were differentiated into macrophages using 50 ng/mL PMA for 48 hours and then rested in PMA-free medium for an additional 24 hours before use.

HEK 293T cell line (ATCC CRL-3216) was cultured in DMEM with 10% FBS, 100 U/mL penicillin, and 0.1 mg/mL streptomycin at 37°C in a humidified atmosphere with 8% CO_2_. Cells were detached using trypsin and split every 3 days.

THP-1 *Renilla*-sg cells were obtained from Dr. Richard Kumaran Kandasamy to be used as a control. These cells were stably transduced with the lentiCRISPR v2 viral construct carrying sgRNA targeting the firefly *Renilla* gene that does not exist in the human genome.

### Generation of *CD5L*-knockout and *RORA*-knockout lines

Three sgRNAs were designed for each gene using the Broad Institute sgRNA design tool (https://portals.broadinstitute.org/gpp/public/analysis-tools/sgrna-design). The targets are listed in **Table 1**.

**Table 1:** sgRNAs designed to induce mutation in THP-1 cells.

CD5L targets (5′–3′)	RORα targets (5′–3′)
G1: GGCTCATACAAAATACCACT, exon 3	ACCATCTCGAGACATCCCTA, exon 4
G2: CCCAGAAGGGCAATCCTGAA, exon 4	AGTTGGGGAAGTCTCGCCGT, exon 5
G3: TCTGGCACACGGTATACCAC, exon 4	GACGCCCACCTACAACATCT, exon 5

Oligonucleotides were synthesized and cloned into the lentiCRISPR v2 vector (52961, Addgene) individually by following the oligo cloning protocol (37, 38). Three targeting clones were validated by sequencing. Lentiviral particles were then generated according to the Addgene protocol (http://www.addgene.org/tools/protocols/plko/#E). In brief, 2.5 × 10^6^ HEK 293T cells were seeded in 10-cm dishes. Cells were transfected the day after seeding with 2.4 μg of the psPAX2 plasmid (12260, Addgene), 1.2 μg of the pCMV-VSV-G plasmid (8454, Addgene), and 2.4 μg of the lentiCRISPR v2-CD5L sgRNA plasmids/lentiCRISPR v2-RORα sgRNA plasmids (0.8 μg of each construct) using GeneJuice transfection reagent (70967, Merck). Media were refreshed 12–15 hours post-transfection. Virus-containing supernatant from each knockout (KO) group was collected at days 4 and 5 and stored at −20°C for further use.

A total of 0.5–1 × 10^6^ THP-1 cells in 0.5 mL full THP-1 media were transduced with 1.5 mL of each virus-containing supernatant using 6 μg/mL of polybrene. Cells were incubated overnight and resuspended in fresh media containing 2 μg/mL puromycin. After 7 days of selection, cells were subjected to clonal expansion, and clones were obtained 40–50 days post-transduction. Four independent clones were selected for future analysis: two replicates of Clone 1 (samples C12 and C12), Clone 2 (C2), and Clone 3 (C3). The presence of deletions in selected clones in targeted exons 3 and 4 groups was confirmed via sequencing (**Supplementary Figure S1A**). A frameshift mutation in RORA exon 4 is shown in **Supplementary Figure S1B**.

### Generation of *CD5L*/*RORA*-double knockout line

The *RORA*-KO clone was used to generate a double knockout (DKO) line. CD5L-targeting oligonucleotides were synthesized and cloned into the lentiCRISPR v2-Blast vector (83480, Addgene) individually, and three validated targeting clones were used to generate the DKO mutant as described before (**Supplementary Figure S1C**).

### RNA isolation and quantitative real-time PCR

Total cellular RNA was isolated by RNeasy Mini kit (74106) with DNAse I digestion (79254) using the QIAcube instrument (all from Qiagen) according to the manufacturer’s instructions. Relative quantifications were performed via quantitative real-time RT-PCR using the StepOnePlus PCR system, TaqMan probes, and TaqMan™ RNA-to-CT™ 1-Step Kit (4392938, Thermo Fisher Scientific) according to the manufacturer’s protocol. The probes used were TNF, Hs00174128_m1; IL-6, Hs00985639_m1; IL-1β, Hs00174097_m1; and GAPDH, Hs99999905_m1. Gene expressions were normalized to GAPDH as an endogenous control, and relative expression values were calculated as fold induction over controls.

### Western blotting

Cells were lysed in a Radioimmunoprecipitation Assay buffer (RIPA) buffer (Sigma Aldrich, MS, USA) that included 1× protease inhibitor cocktail (Sigma Aldrich, P8340, 1/100). Lysates were cleared by 15-min centrifugation at 12,000 *g* at 4°C. Equal amounts of protein (40 μg) were mixed in 2× LDS Sample Buffer (NuPage^®^, Invitrogen) containing 50 mM DTT and denatured for 10 minutes at 95°C, followed by 1-minute sonication and 10-minute centrifugation at 10,000 *g*. Cellular proteins were separated via electrophoresis in 4%–12% Bis–Tris mini protein gels (NuPage^®^, Invitrogen) and transferred to Polyvinylidene Fluoride (PVDF) membranes by electroblotting (iBlot 2 Gel Transfer Device IB21001, Thermo Fisher Scientific). Non-specific protein binding sites on the blots were blocked by a 1-hour incubation with 5% non-fat dry milk solution in Tris-buffered saline supplemented with 0.01% Tween 20 (TBST). The primary antibodies against CD5L (mouse monoclonal F1 clone, 1/1,000, sc-514283, Santa Cruz Biotechnology), GAPDH (1/2000, sc-47724, Santa Cruz Biotechnology, Santa Cruz, CA USA), RORA (1/1,000 PP-H3910-00, R&D Systems), and the HRP-conjugated goat anti-mouse secondary antibody (1/5000, #P0447, Dako) were diluted in 0.5× blocking buffer/PBS. The bound Horseradish Peroxidase (HRP)-conjugated secondary antibodies were visualized using the SuperSignal West Femto Maximum Sensitivity Substrate (Thermo Fisher Scientific). Proteins were detected and imaged using an Odyssey^®^ Fc Imager (926-40020, Li-Cor Biosciences).

### ELISA

The levels of secreted inflammatory cytokines in the supernatants of control and LPS-stimulated cells were quantified using R&D Systems DuoSet ELISA (DY210 for human TNF and DY206 for human IL-6) per manufacturer’s instructions.

### RNA-seq analysis

Total RNA was isolated from duplicates of differentiated KO and control cell lines. RNA quality control was performed using the Agilent 2100 Bioanalyzer System. Libraries were prepared at Novogene and sequenced on an Illumina instrument (**Supplementary Table S1**; Novogene, Hong Kong) as 150-bp paired-end reads. Sequences were aligned to the human genome (GRCh38.hg38) using HISAT2 v2.1 graph-based aligner. Aligned gene reads were imported into the Partek Genomics Suite software package and annotated using the hg38 RefSeq transcript database. All subsequent quantifications and analyses of data were performed in Partek Genomics Suite. All significance cutoffs were adjusted for false discovery rate (FDR). Gene Ontology (GO) and KEGG pathway enrichments were performed using the Partek Genomics Suite with built-in functions. The heat maps of gene reads were generated using the Broad Institute online Morpheus tool (https://software.broadinstitute.org/morpheus/).

### Lipidomics

A total of 10^7^ cells were seeded in 10-cm culture dishes using 50 ng/mL PMA for 48 hours. Total lipid was extracted after 24 hours of incubation in PMA-free media. Media were discarded, and 1 mL of cold isopropanol was added to the dishes. Dishes were then vortexed for 30–60 seconds, and cells were scraped off and subjected to centrifugation at 4°C with maximum speed for 5 minutes. Supernatants were stored at −20°C prior to Ultra-Performance Liquid Chromatography -Electrospray Ionization - Time-of-Flight (UPLC-ESI-TOF) mass spectrometry analysis at VITAS AS, Oslo, Norway. All lipid isolation steps were performed on ice. All samples were prepared in duplicates.

### Filipin staining

A total of 8 × 10^4^ cells were differentiated using 50 ng/mL PMA in a μ-slide 8-well ibiTreat plate (80826, Ibidi, Gräfelfing, Germany). Twenty-four hours after being rested in PMA-free media, cells were fixed with 3% formaldehyde for 30 minutes. Cells were then washed twice with PBS and were stained with a stock solution of Filipin dye (Filipin III from *Streptomyces filipinensis*, F4767, Sigma Aldrich) with a concentration of 50 μg/mL in 10% FBS–PBS for 1 hour. Cells were washed three times with PBS, and microscopy was performed using an Olympus IX71 fluorescent microscope. Quantitative estimates of fluorescent signal strength are provided as Corrected Total Cell Fluorescence (CTCF) values: CTCF = Integrated Density − (Area of selected cell × Mean fluorescence of background).

### FASN activity assay

A total of 10^6^ cells A total of were lysed in 200 μL lysis buffer (7 mM HEPES, 320 mM sucrose, and 1 mM DTT, pH 7.4) with one protease inhibitor tablet per 50 mL of buffer. Lysates were then immediately frozen at −80°C and subsequently thawed on ice. Thawed lysates were homogenized using a probe sonicator (15%–20% power, Branson Sonifier 450 Digital Ultrasonic Cell Disruptor) for 3 pulses of 2 seconds with 1-minute intervals on ice. Homogenized lysates were centrifuged at 4°C at maximum speed for 10–15 minutes. Cleared supernatants were used to measure protein concentration. The activity assay was performed in a 96-well microplate. One hundred microliter activity assay buffer (200 mM potassium phosphate, pH 6.6, 1 mM, 1 mM EDTA, 0.24 mM NADPH, and 30 μM acetyl-CoA) was added to 70 μg of each sample supernatant. For blank, an equal volume of activity assay buffer and lysis buffer was used. OD_340nm_ was monitored for 3 minutes to measure background NADPH oxidation (POLARstar OMEGA plate reader, BMG LABTECH). Malonyl-CoA was added at a final concentration of 50 μM to the samples and blank, and OD_340nm_ was monitored for 15 minutes to determine FASN-dependent NADPH oxidation. All measurements were performed in duplicates. The rate change of OD_340nm_ was corrected for the background rate of NADPH oxidation. The unit of FASN enzyme activity was defined as the amount of enzyme that oxidizes 14 nmol NADPH to NADP and produces 1 nmol palmitate in 1 minute. The unit of FASN in a 1-mL solution was calculated using the following formula:


$$\text{Activity }\left(\text{U}/\text{mL}\right)\;=\;{\text{A}}_{340}/\text{e }\times \text{ V }\times \;10^{6}\times \text{ C}/14,$$

where A is the decrease of absorbance at 340 nm in 1 minute; e is the mmol extinction coefficient, which is 6.022 in a 1-cm light path; V is the assay mixture volume in liters; and C is the dilution factor.

### RORE reporter assays

The hSPARC-Tk-Luc reporter was created by cloning oligonucleotides composed of three copies of the human SPARC promoter ROR response elements (ROREs) in the sense-sense-sense orientation (5′-GATCTGCTGTTCTGGGTCATCCCGCTGTTCTGGGTCATCCCGCTGTTCTGGGTCATCCCA-3′) into the *Bgl*II restriction site in the TkpGL3 firefly luciferase reporter vector (Promega, Madison, WI, USA) as described by Chauvet et al. (39). The same oligonucleotide cloned in the inverted position was used to create the negative control construct (mutSPARC). Transfection efficiency was controlled by co-transfection of the TK-Nanoluc control vector at a 1:10 ratio. Luciferase expression by the reporter and control constructs at 48 hours post-transfection was quantified using Nano-Glo^®^ Dual-Luciferase^®^ Reporter Assay (Promega).

### Statistical analysis

Statistical analysis for experiments consisting of a minimum of three independent biological replicates was calculated using two-tailed Student’s *t*-test within the GraphPad Prism software package for all experiments unless stated otherwise in the figure legends. Differences with *p* < 0.05 were considered statistically significant. Column figures are presented as means ± SEM.

## Results

### CD5L controls inflammatory state of differentiated THP-1 cells

**Figure 1:**
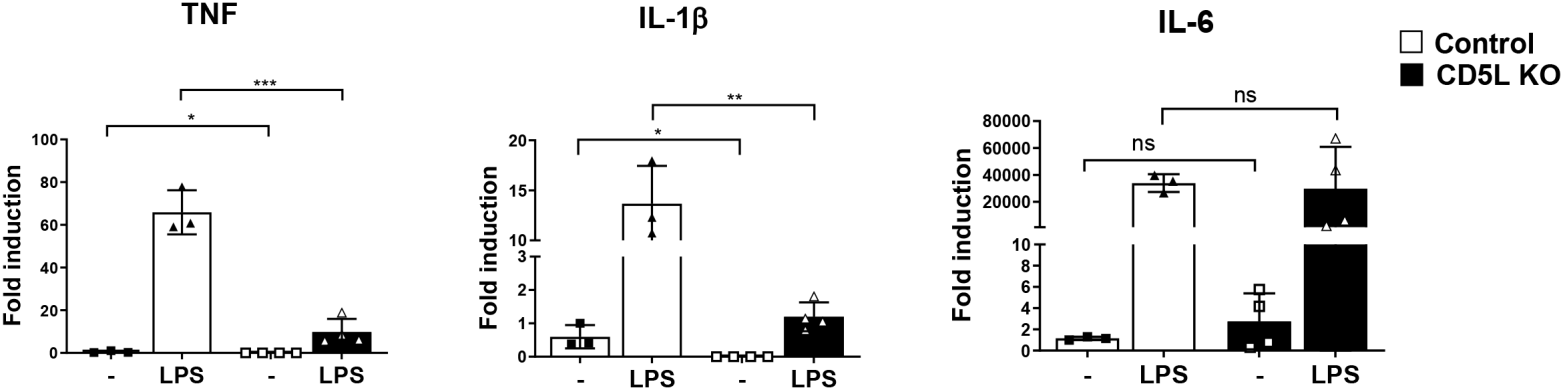
*CD5L*-KO cells have reduced inflammatory state. Deletion of *CD5L* led to decrease in basal and LPS-induced cytokine mRNA levels. *TNF* and *IL-1β* levels in unstimulated cells were decreased nearly 300-fold, with average basal levels of 0.003 for knockout cells compared to the value of 1 set for the controls. A total of 2.5 × 10^5^ cells in 24-well plates were differentiated using 50 ng/mL PMA for 48 hours. Stimulation was performed after 24 hours of rest in PMA-free media with 100 ng/mL LPS for 4 hours. Inflammatory cytokine expression levels were determined using qRT-PCR of RNAs isolated from cell lysates. Graphs are representative of at least three independent experiments. PMA, phorbol 12-myristate 13-acetate. (* p<0.05, ** p<0.01, *** p<0.001).

**Figure 2:**
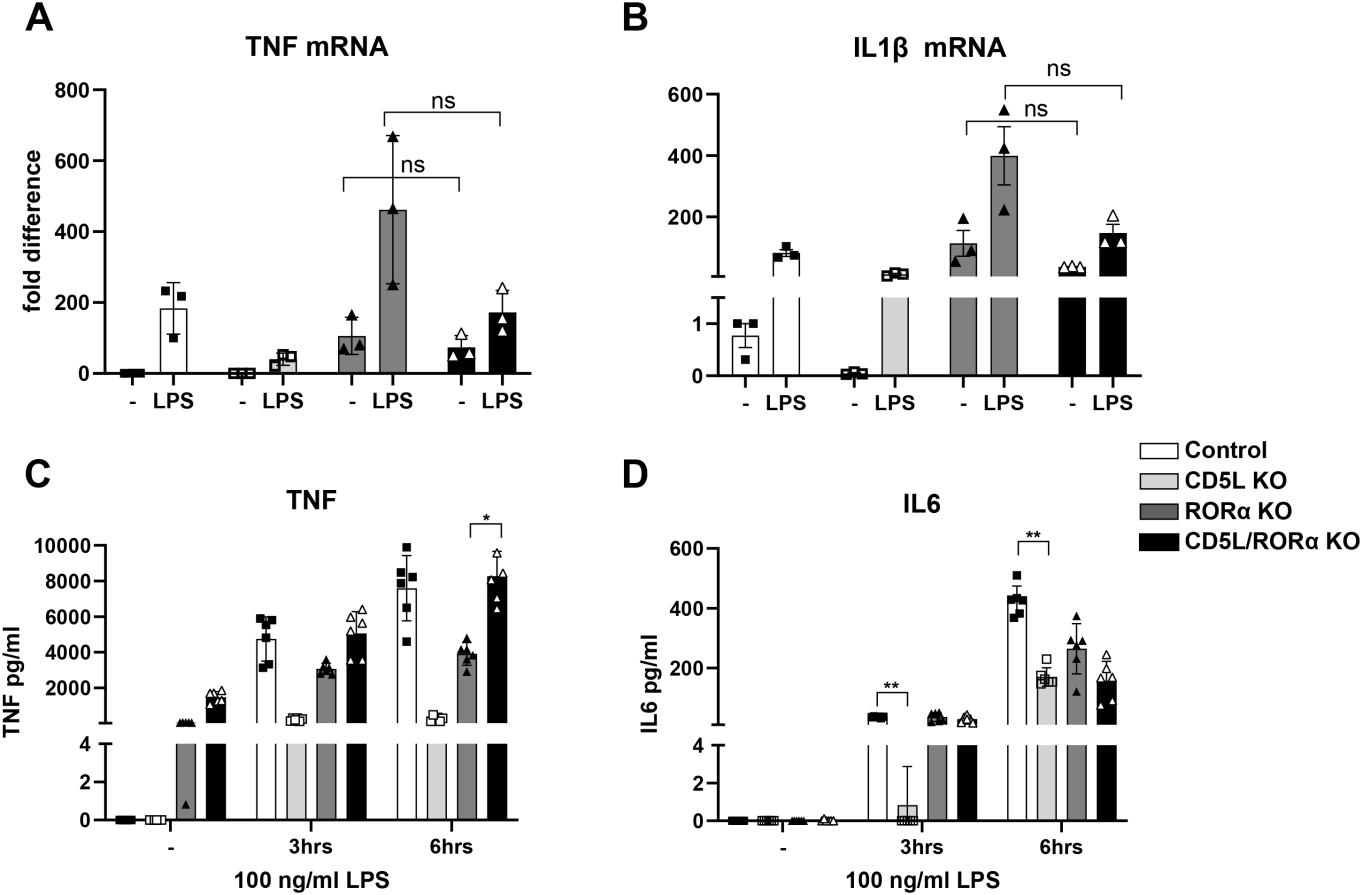
*RORα* is epistatic to *CD5L*. Induction of inflammatory TNF **(A)** and IL-1β **(B)** gene expression in *CD5L/RORA* DKO cells is similar to that observed in *RORA* deletion cells (n = 3, ± SEM). Paired two-tailed *t*-test of three biological replicates has been used to evaluate statistical significance of differences between *RORAko* and *CD5L/RORA* DKO cell lines. Both *RORAko* and *CD5L/RORA* DKO cells produce similarly higher levels of TNF than the *CD5Lko* cells at 3 and 6 hours after LPS stimulation **(C)**. *CD5Lko* cells produce significantly lower amounts of IL-6 at both the 3- and 6-hour timepoints, while *RORAko* and *CD5L/RORA* DKO secrete similar levels of this cytokine **(D)** (n = 6, ± SEM, representative of two independent experiments * p<0.05, ** p<0.01).

CD5L involvement in the control of inflammatory signaling is well documented (6, 40). Nevertheless, there is some disagreement about its impact on the inflammatory function at the cellular and systemic levels. Sanjurjo et al. showed the anti-inflammatory role of CD5L in THP-1 cells that stably overexpressed *CD5L* (19). However, several recent works also provide support for a pro-inflammatory role of CD5L in mouse models (20, 21) as well as human chondrocytes [Brigant et al., submitted]. We therefore chose to determine the role of CD5L in the control of inflammatory signaling in human macrophages. To achieve this, we analyzed the effect of disrupting the *CD5L* gene in the widely used THP-1 monocytic cell line differentiated into macrophage-like cells by treatment with PMA (41). We used a lentiCRISPR v2-based lentiviral delivery system (37, 38) to simultaneously introduce *CD5L*-targeting guide RNAs and Cas9 nuclease into THP-1 cells. Following the selection of transduced cells and single-cell cloning, we confirmed the deletion of both *CD5L* alleles by sequencing (**Supplementary Figure S1A**). When compared to control cell lines that were transduced with the lentiviral construct carrying sgRNAs targeting *Renilla* luciferase, we found that the deletion of *CD5L* in THP-1 cells has altered their basal inflammatory state (**Figure 1**). Even in the absence of additional stimulation, we saw that the expression levels of *TNF* and *IL-1β* inflammatory cytokines were dramatically lower in *CD5L*-KO cells compared to controls. Exposure of mutant cells to 100 ng/mL of K12 LPS (InvivoGen) induced the mRNA levels of these cytokines in both the mutant and control cells. However, in CD5L mutants, the levels of LPS-induced transcripts remained significantly lower than those in the control cells post-stimulation. While the magnitude of induction was similar in both mutant and control cells, *CD5L* deletion did not affect IL-6 pro-inflammatory cytokine expression at this timepoint. Subsequent ELISA quantification of secreted IL-6 revealed that *CD5L* mutant cells produced significantly lower levels of this cytokine at the 3- and 6-hour timepoints after LPS stimulation (**Figure 2D**). Together, these observations of reduced inflammatory cytokine expression in cells lacking *CD5L* support the pro-inflammatory role of this gene in differentiated human monocytes.

### Deletion of *CD5L* remodels transcriptome of macrophage-like cells

**Figure 3:**
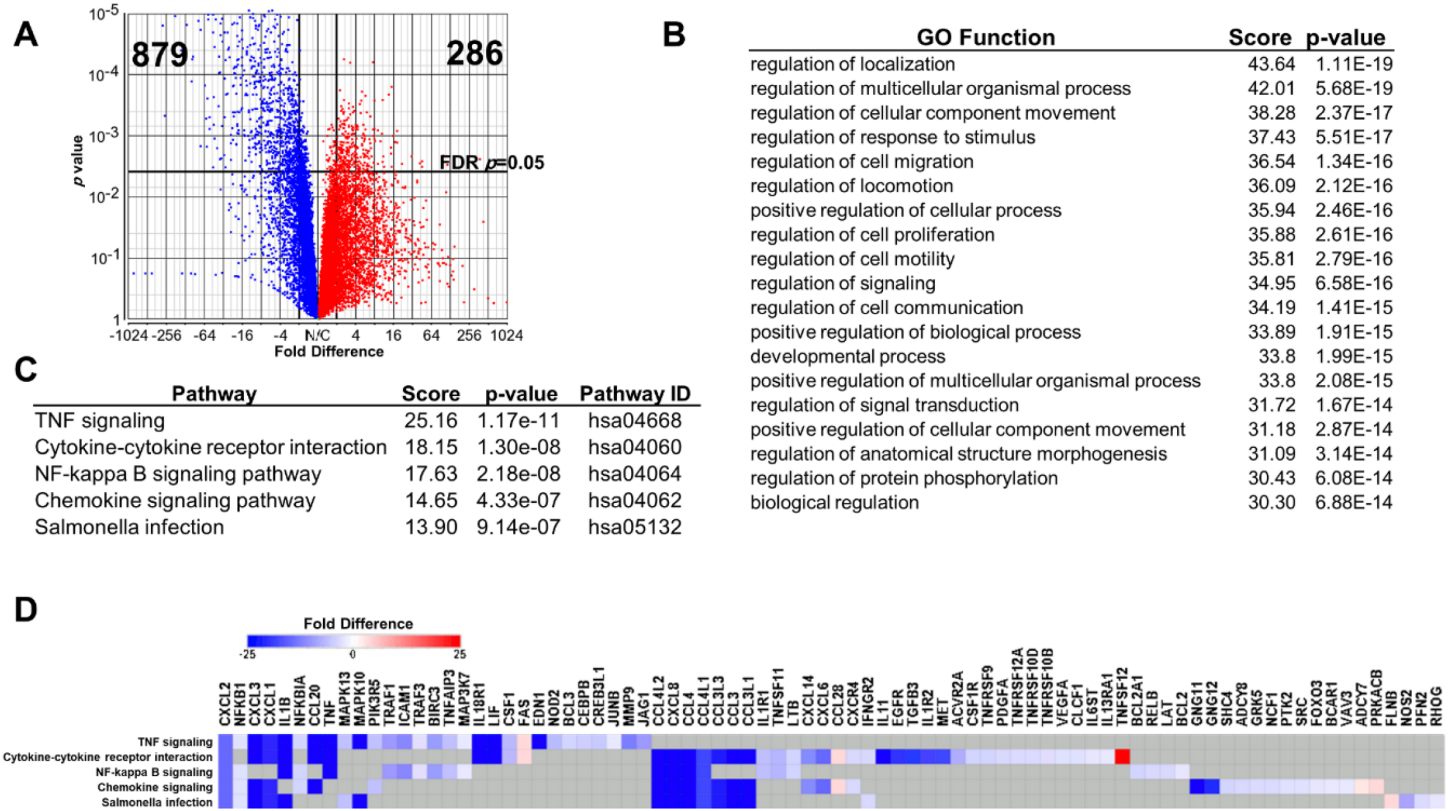
RNA-seq analysis of differentiated *CD5L* deletion cells. **(A)** Volcano plot of ANOVA results. Vertical cutoff line is at FDR-adjusted *p* = 0.05. Vertical cutoff lines are at fold difference = 2. A total of 1,165 annotated genes were found to be differentially expressed between *CD5L* deletion and control cell lines using these criteria. The majority of differentially expressed genes (879) were downregulated (blue). **(B)** Gene Ontology terms enriched by the differentially expressed dataset include regulation of intracellular processes as well as cell-to-cell signaling characteristic of immune cells. **(C)** All five most significantly enriched KEGG pathways mediate immune signaling. **(D)** The magnitude of changes in expression levels of genes that contribute to the top five enriched KEGG pathways. For genes that are not part of a specific pathway, the values are shown in gray. Colors represent fold difference change in gene expression when compared to controls. FDR, false discovery rate.

To determine the extent of transcriptome changes induced by the deletion of *CD5L*, we used RNA-seq to compare the levels of gene expression in four isolates [two replicates of Clone 1 (samples C12 and C12), Clone 2 (C2), and Clone 3 (C3) from two independent targeting experiments] of mutant unstimulated differentiated THP-1 cell lines and two control cell lines. We identified 1,165 genes that were differentially expressed more than twofold when using a stringent FDR-adjusted *p* < 0.05 cutoff for significance (**Figure 3A**). A total of 879 genes out of 1,165 were downregulated, and 286 genes were upregulated in mutant cells. In this differentially expressed dataset, all three sub-ontologies (Biological Process, Molecular Function, and Cellular Component) were similarly represented (enrichment scores ranging from 6.21 to 7.63). The majority of the terms with the highest scores (**Figure 3B**) were contained within the Regulation of Biological Process term (GO: 0050789). We further confirmed the global impact of *CD5L* deletion on inflammatory signaling via KEGG pathway enrichment analysis, which revealed that most inflammatory signaling pathways are significantly impacted by the deletion of *CD5L* (**Figure 3C**). Consistent with our observations, TNF signaling, cytokine–cytokine receptor interaction, and NF-kappa B signaling pathways were the most significantly enriched by our differential expression dataset. A total of 82 of the differentially expressed genes (**Figure 3D**) were annotated with the top five significantly enriched pathways (**Figure 3C**), including *TNF* and *IL1B* identified in our initial analysis (**Figure 1**).

**Figure 4:**
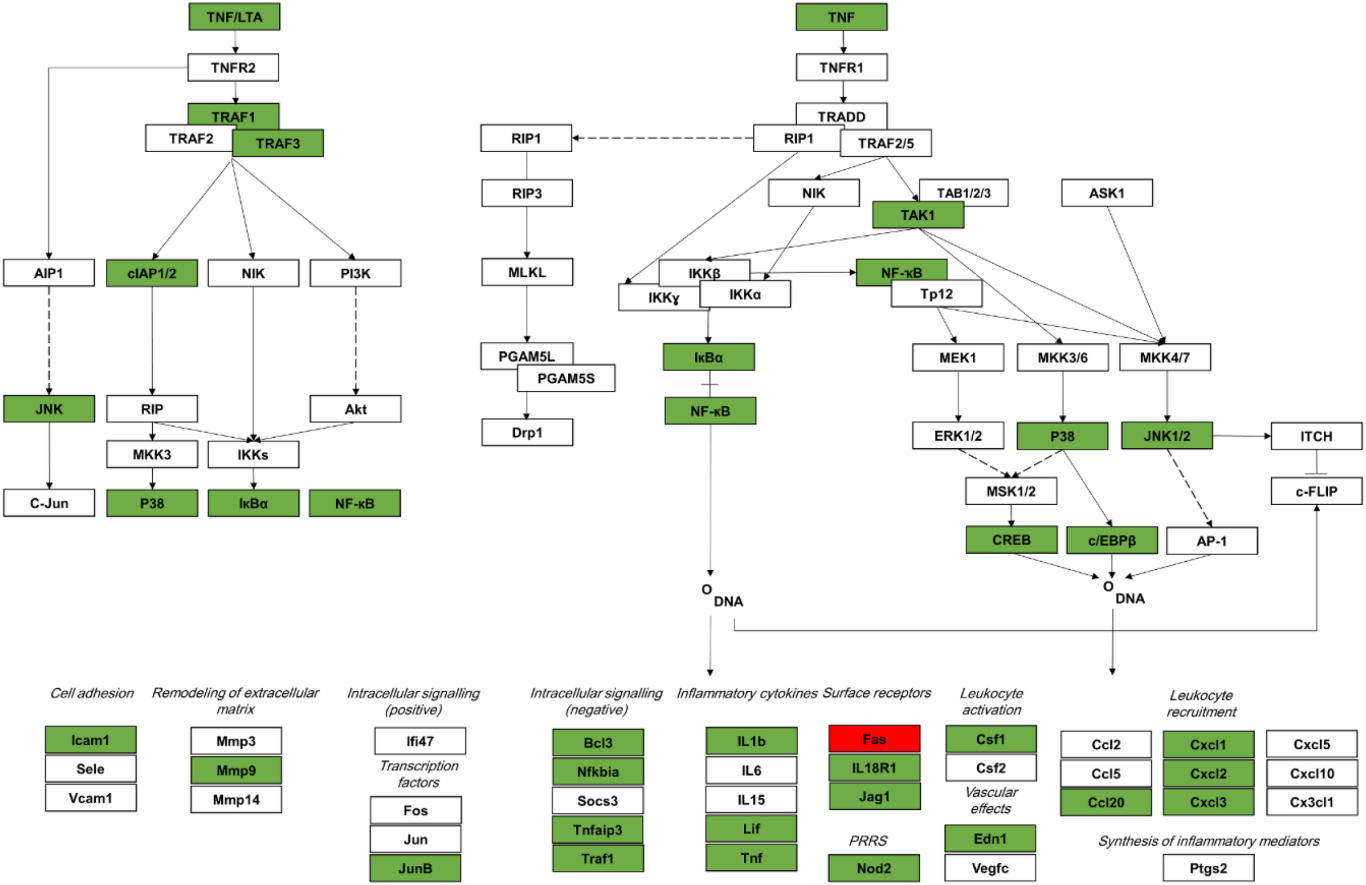
Expression of genes in the TNF signaling pathway is impacted by deletion of *CD5L*. Deletion of *CD5L* results in downregulation (green) of transcription of nearly half of the genes comprising the TNF signaling pathway. Expression of signal propagating kinases is also affected by p38, and JNK is downregulated at the transcriptional level (adapted from KEGG TNF signaling pathway, hsa04668 6/25/18).

A total of 28 members of the TNF signaling KEGG pathway were downregulated (**Figure 4**) in *CD5L* deletion cells, and only one (FAS/TNFRSF6) was upregulated. Taken together, our data suggest that CD5L is responsible for the maintenance of the inflammatory baseline in human macrophages, and in its absence, cells enter the constitutive hypo-inflammatory state.

### CD5L remodels lipidome in THP-1 cells

**Figure 5:**
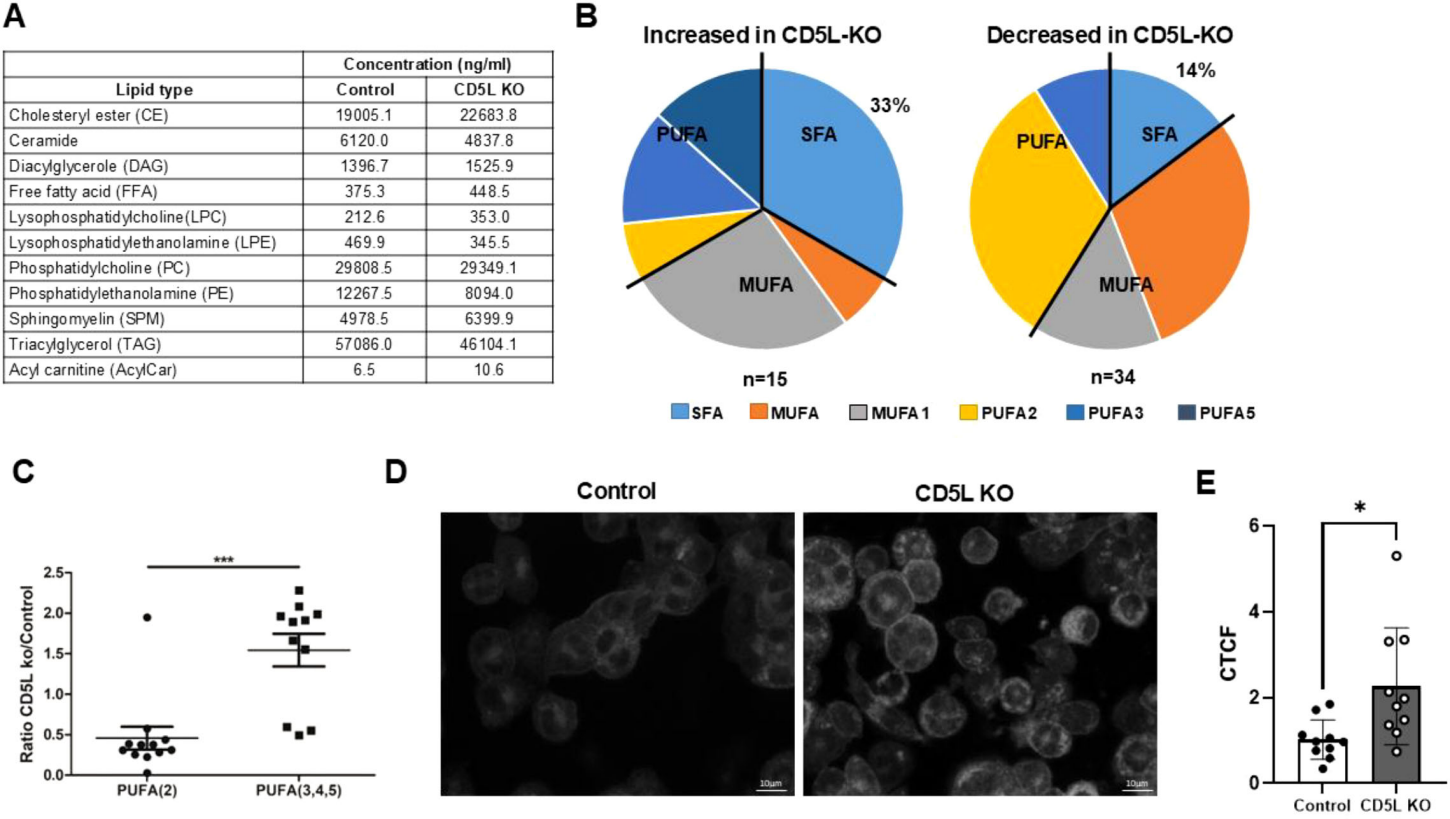
Lipid remodeling function of CD5L. **(A)** Total levels of lipid classes analyzed in mutant and control cells. **(B)** Proportion of side acyl chains classified according to the extent of their saturation. MUFA: one or two double bonds in a 3-acyl chain lipid or one double bond in a 2-acyl chain lipid. MUFA1: number of double bonds and acyl chains is equal. PUFA2, PUFA3, and PUFA4: number of double bonds is two-, three-, and fourfold higher compared to number of acyl chains in the lipid, respectively. **(C)** Deletion of *CD5L* significantly (*** *p* < 0.001, Mann–Whitney test) increases levels of highly polyunsaturated [PUFA(3,4,5)] fatty acids. **(D)***CD5L* deletion results in an increase of free cholesterol in mutant cells. PUFA, polyunsaturated fatty acid. **(E)** Corrected Total Cell Fluorescence (CTCF) values calculated for 10 randomly selected cells for each cell line (* *p =* 0.014 unpaired two-tailed *t*-test).

It has been reported that CD5L is able to alter lipid content in murine adipocytes and Th17 cells (6, 13). Thus, we aimed to test whether CD5L functions in a similar fashion in human macrophages. To achieve this, we performed mass spectrometry-based UPLC–ESI–TOF analysis of total cellular lipids isolated from differentiated mutant and control cells. This approach allowed us to measure the abundance of acyl chains in 11 lipid classes (**Figure 5A**). We found that when compared to control cells, in *CD5L*-KO cells, 33% of the saturated fatty acyl (SFA) side chains were increased and 14% were decreased across the analyzed lipid classes (**Figure 5B**). We also observed that the deletion of *CD5L* increased the levels of highly polyunsaturated fatty acid (PUFA) side chains (**Figure 5C**). Staining for free cholesterol using Filipin dye also revealed an increase in accumulated free cholesterol in mutant cells (**Figure 5D**). Taken together, all these observations demonstrate that endogenous CD5L participates in defining the lipid content of human macrophages.

### FASN activity is not altered in *CD5L* deletion cells

**Figure 6:**
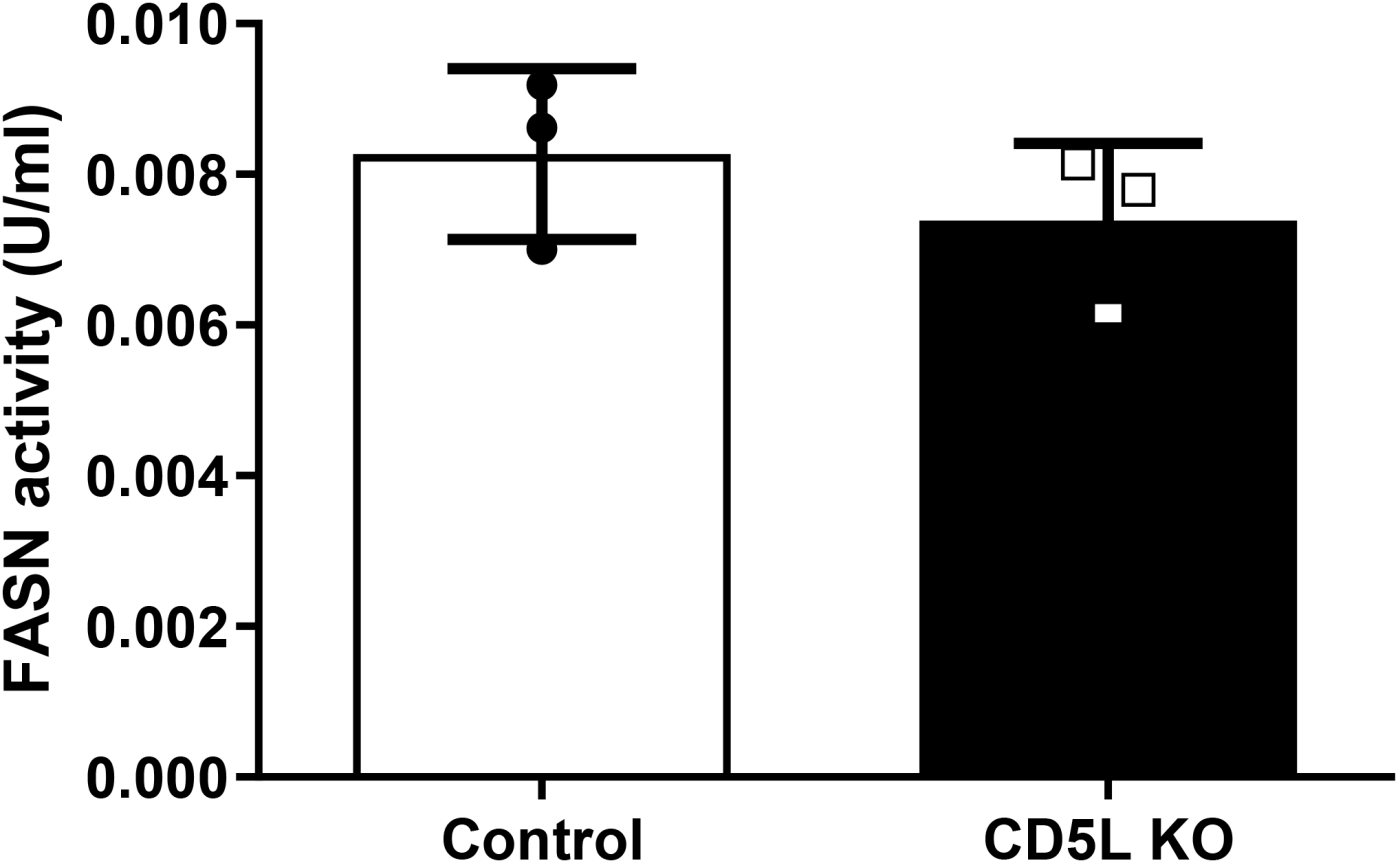
FASN activity in knockout and control cells. Deletion of *CD5L* does not significantly change the overall FASN activity of human macrophages. Lysates from 10^6^ cells were used to measure FASN activity by monitoring NADPH oxidation over 15 minutes. Activity is presented as an average of replicates from three independent experiments. FASN, fatty acid synthase.

A recent study of murine adipocytes has implicated *Cd5l* in the regulation of lipid homeostasis through its binding and the inhibition of FASN (13). To test if CD5L functions in a similar fashion in human macrophages, we used the NADPH oxidation assay to measure FASN activity in mutant and control cells. As shown in **Figure 6**, we found that *CD5L* deletion does not result in significant changes in the overall FASN activity. This suggests that there may be other targets of CD5L that mediate its lipidome remodeling function in human macrophages.

### *RORA* is epistatic to *CD5L*

A recent single-cell analysis of murine Th17 cells revealed that the inflammatory profile of individual cells is determined by the endogenous expression of *Cd5l*. It has been proposed that in these Th17 cells, the presence or absence of *Cd5l* expression determines the ligand availability for the RORγt nuclear receptor transcription factor (6). Human macrophages do not express RORγt but produce RORα, a closely related member of the ROR transcription factor family (26). We hypothesized that in macrophages, the lipidome remodeled by CD5L can be sensed by RORα, similarly to RORγt in Th17 cells. Our earlier work has established that the deletion of *RORA* leads to an elevated basal inflammatory state of THP-1 cells (36). Our observation of increased inflammatory cytokine production in cells lacking *RORA* is opposite to the suppressed state of inflammatory signaling found in *CD5L* mutants. If CD5L-induced changes in macrophage inflammatory state require *RORA*, we could then expect that the deletion of *RORA* in *CD5L* mutant cells would result in the same phenotype as the *RORA* deletion cells. To test this hypothesis, we used CRISPR Cas9 to disrupt exons 4 and 5 of *RORA* in *CD5L*-KO cells (**Supplementary Figure S1C**). We then measured the expression levels of *TNF* and *IL-1β* in THP-1 cells deleted for both *CD5L* and *RORA* genes. As shown in **Figure 2**, *RORA* and DKO cells show a similar hyper-inflammatory phenotype. Both cell lines that lack *RORA* secrete detectable amounts of TNF even in the absence of stimulation (**Figure 2C**) and accumulate similar levels of both TNF and IL-6 at multiple timepoints after LPS exposure. This observation suggests that *RORA* is epistatic to *CD5L* and therefore supports our hypothesis that CD5L’s role in the inflammatory state of macrophages is mediated by RORα.

### A subset of genes is divergently regulated by CD5L and RORA

**Figure 7:**
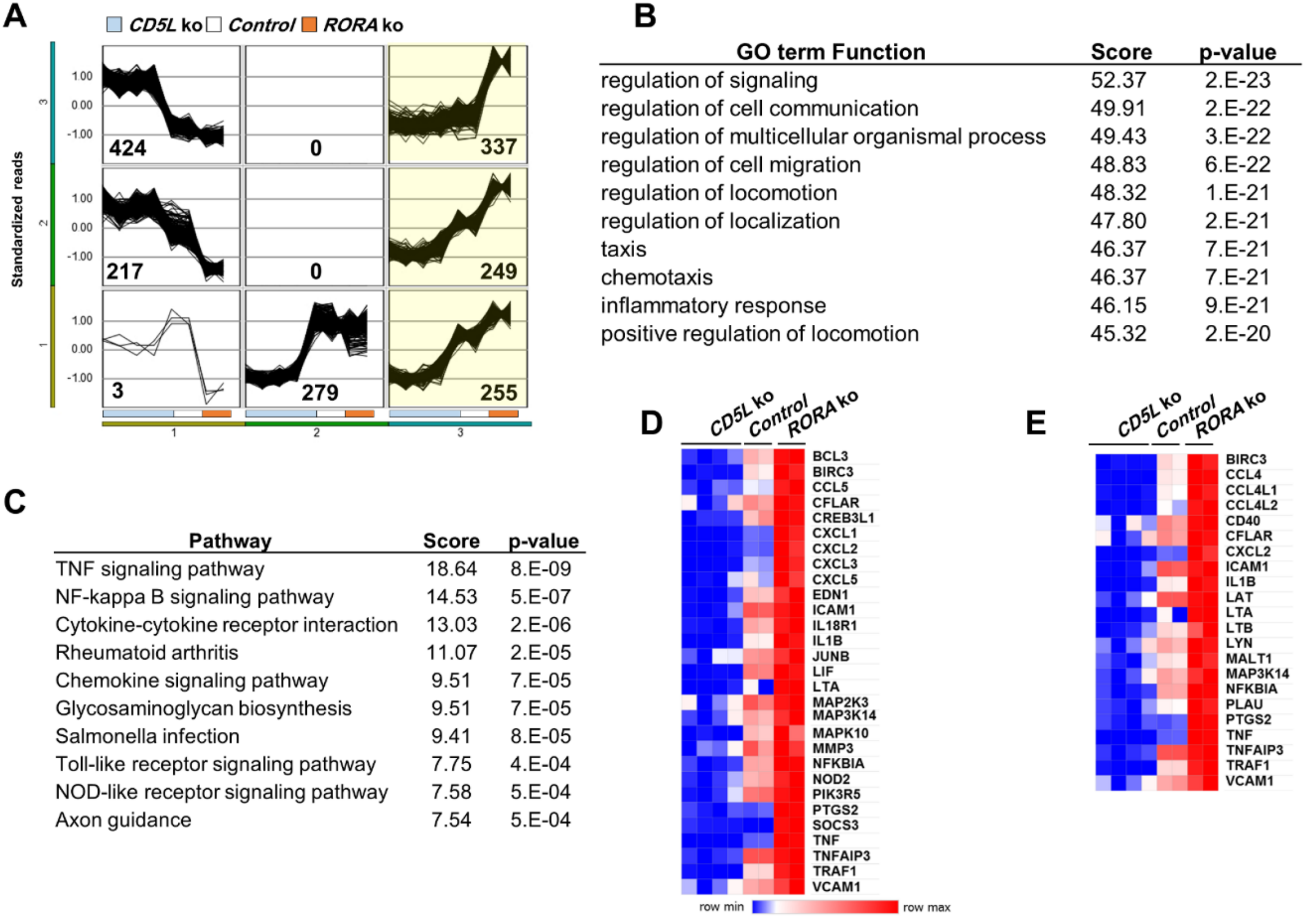
Differences in gene expression in *CD5L* and *RORA* deletion cells. **(A)** Self-organizing map (SOM) clustering identified six distinct patterns of changes in gene expression between respective mutant and control cells. **(B)** GO term analysis of genes in highlighted divergently regulated clusters identifies terms characteristic of immune response. **(C)** Eight out of 10 KEGG pathways enriched by genes from highlighted clusters are central to shaping the inflammatory signaling of immune cells. Genes contributing to the TNF **(D)** and NF-κB **(E)** signaling pathways are downregulated in *CD5L* mutant cells and upregulated in *RORA* mutants. GO, Gene Ontology.

To provide further support for our hypothesis, we extended our RNA-seq analysis to incorporate data from the *RORA* deletion cell line (36). We found 1,751 genes to be differentially expressed more than twofold with the stringent, FDR-corrected *p*-value of 0.01 in *CD5L* and *RORA* deletion cells. Self-organizing map (SOM) clustering of genes in this dataset identified six major clusters (**Figure 7A**) (42). Clusters 3, 6, and 9 (highlighted) consisted of genes that were upregulated in *RORA* deletion cells and downregulated in *CD5L* knockouts. GO terms characteristic of immunoregulatory processes were the most enriched in this divergently regulated subset of genes (**Figure 7B**). Furthermore, KEGG TNF and NF-κB signaling pathways were the most significantly enriched by genes forming these clusters (**Figure 7C**). The majority of genes in TNF and NF-κB signaling pathways found to be downregulated in *CD5L* mutant cells were consistently upregulated in *RORA* mutants (**Figures 7D, E**). This observation provides further support for our hypothesis that CD5L’s control of inflammatory signaling is at least in part controlled by RORα.

### CD5L deletion does not change expression of RORE-driven reporter

RORα drives the expression of its effectors by binding to relatively well-defined ROREs. Therefore, we hypothesized that a definitive proof of CD5L involvement in the regulation of RORα transcriptional activity can be obtained by comparing the expression of RORE-driven reporters in cells with and without CD5L. To test this, we created a reporter that had three tandem ROREs in the sense position in front of the thymidine kinase minimal promoter-driven firefly luciferase. An identical reporter with a similarly sized insert was used as a negative control. The induction of luciferase from the sense and not the control reporter was confirmed via the transfection of 250 ng of a construct constitutively expressing the human *RORA* gene. Following the transfection of 3xRORE RORE reporters into both THP-1 and HEK293 cell lines that were wild type or deleted for CD5L, we found no changes in the reporter gene expression that can be attributed to the presence of endogenous CD5L (data not shown). Therefore, at present, we cannot conclude that in unstimulated cells, the presence of endogenous CD5L controls the basal transcription activity of RORs.

### CD5L deletion dysregulates expression of factors controlling cell-to-matrix interactions

**Figure 8:**
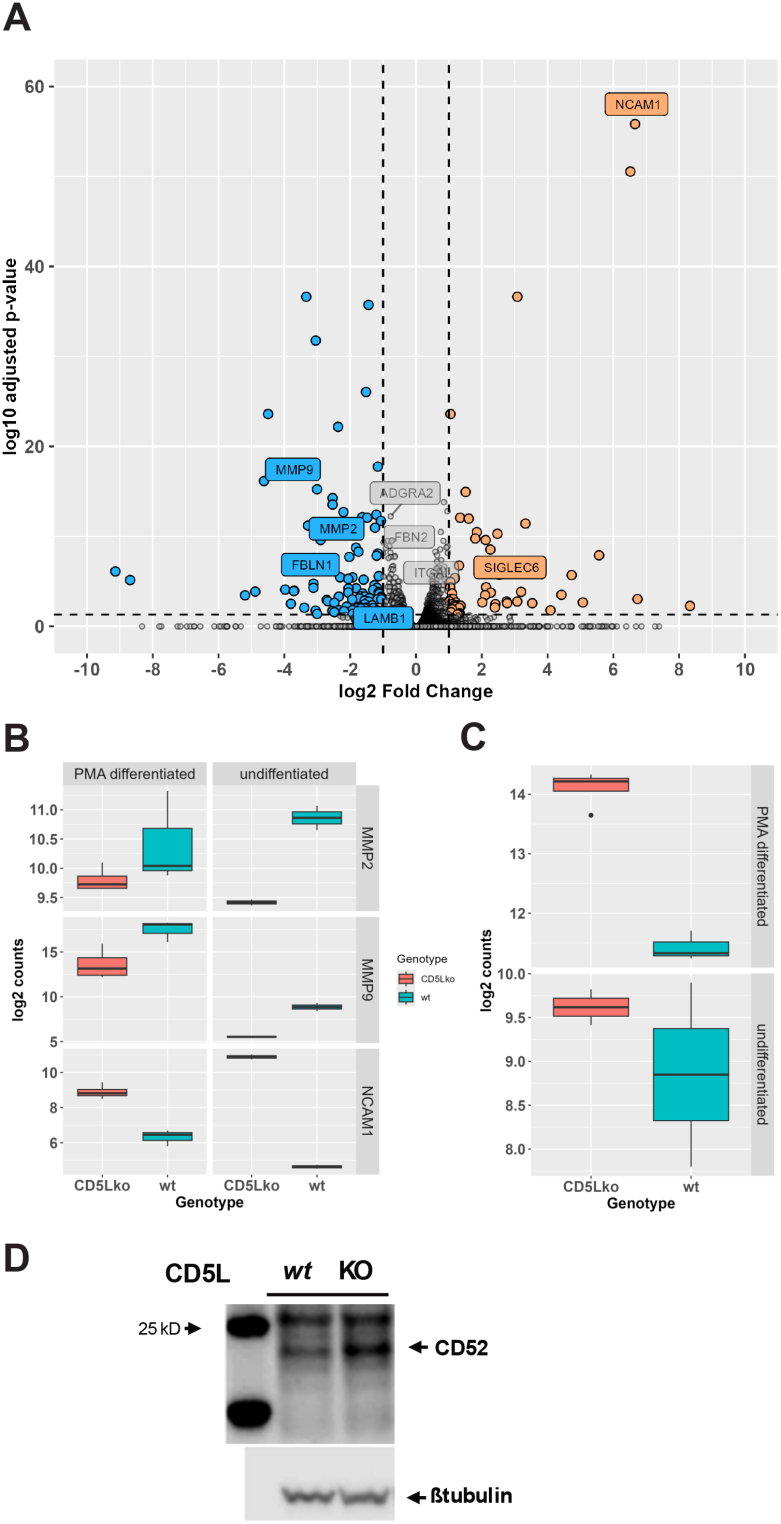
CD5L deletion induces transcriptional changes in undifferentiated monocytes. **(A)** Deletion of CD5L in undifferentiated monocytic THP-1 cells significantly changed expression of 52 genes more than twofold up and 95 genes twofold down (in color). **(B)** Expression of atherogenesis-related genes involved in interaction of monocytes with ECM is changed in CD5Lko cells. **(C)** CD52 expression is significantly increased in undifferentiated (FDR = 3e−14) and differentiated (FDR = 2.9e−35) CD5Lko cells. **(D)** Cellular CD52 protein levels are increased in undifferentiated CD5Lko THP-1 monocytes. ECM, extracellular matrix; FDR, false discovery rate.

PMA used for the differentiation of THP-1 monocytes is itself an inducer of inflammation. Therefore, PMA-differentiated macrophages can have inflammation-related remodeling of their transcriptomes even after withdrawal of stimulation, as illustrated by a large number of differentially regulated genes in our RNA-seq dataset described above. This makes it difficult to identify causative events that define the observed suppression of inflammatory signaling in PMA-differentiated CD5L knockouts. Furthermore, in our experiments described above, we used single-cell clones that could fix non-specific changes in gene expression driven by the initial growth in the absence of cell-to-cell interactions (43). To address these shortcomings and to identify basal transcriptome changes that can be directly attributed to CD5L function, we chose to expand our studies to include the analysis of CD5L knockout in unstimulated populations of suspension-grown THP-1 monocytes. To this end, we generated a separate set of knockout cell lines using a new set of targeting and non-targeting guide RNAs (**Supplementary Table S2**). Because the endogenous levels of CD5L are below the threshold of detection by conventional Western blotting (WB), we used an indirect approach to validate the efficiency of selected guides by introducing a cytomegalovirus (CMV) promoter-driven CD5L transgene in cells prior to transduction with the targeting guides. We found that selected guides effectively reduce the levels of exogenous CD5L, allowing us to assume that the endogenous copy is edited efficiently (**Supplementary Figure S3**). Following transduction and selection with puromycin, we isolated total RNA from unstimulated cells in which *CD5L* was targeted by two separate guides and two independent isolates of cells transduced with the non-targeting control. As expected, our analysis of differences in the relative abundance of transcripts in undifferentiated control and mutant populations identified a much smaller set of differentially expressed genes. There were 52 genes significantly (FDR < 0.01) upregulated in *CD5Lko* cells more than twofold, and the expression of 95 genes was reduced more than twofold. Comparison of differentiated and undifferentiated datasets revealed another set of genes whose expression was impacted by the deletion of *CD5L*. Specifically, the transcription of matrix metalloproteases MMP-2 and MMP-9 was reduced, and the expression of neural cell adhesion molecule-1 (NCAM-1) adhesion molecule was increased in cells lacking CD5L (**Figure 8B**). Overall, a common theme for observed changes in the expression of these molecules fits the earlier proposed role of CD5L in the control of atherogenic potential of monocytes. Elevated levels of MMP-2 and MMP-9 are among the main drivers of atherogenesis (44). However, NCAM-1 levels are lower in patients with coronary artery disease (45). Thus, the observed direction of changes in the expression of these three factors controlling cell-to-matrix interactions is consistent with the pro-atherogenic effect of CD5L.

### CD5L deletion induces CD52 suppressor of inflammation

We hypothesized that the primary effects of CD5L deletion on gene expression should reveal themselves in a similar fashion in both differentiated and undifferentiated datasets. Intersection of genes that were upregulated in both datasets identified a small set consisting of 16 genes (**Supplementary Table S1**). In this set, our attention was drawn to *CD52* that encodes a small surface glycoprotein also known as CAMPATH-1. In undifferentiated *CD5Lko* monocytes, *CD52* was induced 3.6-fold (FDR = 3.34e−11), and in differentiated knockouts, its levels were elevated 6.34-fold (FDR = 2.8e−35) (**Figure 8C**). Western blotting of undifferentiated THP-1 cells that were deleted for CD5L revealed elevated levels of cellular CD52 in the knockout cells when compared to the non-targeted guide transduced controls (**Figure 8D**). This induction of CD52 is notable because it has been recently shown that its soluble form inhibits TLR-mediated activation of NF-κB as well as triggers apoptosis (46). This makes CD52 a plausible candidate for a CD5L-dependent regulator of inflammation.

## Discussion

The main finding of our study is that the presence of endogenous CD5L is necessary to maintain the basal inflammatory state in human macrophages. *CD5L* deletion resulted in constitutive downregulation of a subset of NF-κB-regulated genes, including *TNF* and *IL-1β*. Our RNA-seq analysis revealed substantial alterations in the transcriptome of *CD5L*-KO cells that mainly involved genes participating in inflammatory signaling.

Several recent studies have drawn attention to the role of the abundant circulatory scavenger receptor-like molecule CD5L in defining the inflammatory properties of both immune and non-immune cells (6, 19–21). However, it appears that CD5L can impact inflammatory signaling in different directions, depending on the organism and cell type. Our work was designed to address some of the outstanding issues regarding CD5L and to define its function in human macrophages. We pursued this goal by deleting both alleles of *CD5L* in the human monocyte-like THP-1 cell line. We found that the deletion of *CD5L* resulted in decreased expression of pro-inflammatory cytokines at both basal and LPS-stimulated levels when compared to control cells. Reduced basal expression of *TNF* was observed in both undifferentiated and PMA-differentiated *CD5L* deletion cell lines (**Figure 1**, **Supplementary Figure S2**). These observations support the pro-inflammatory function of CD5L in human macrophage-like cells. Even though the basal expression levels of *TNF* and *IL-1β* are lower in mutant cells, in response to LPS stimulation, the magnitude of induction is similar in both mutant and control cells. This suggests that the lack of CD5L did not interfere with the response to LPS. The deletion of *CD5L* did not affect IL-6 cytokine expression levels, suggesting that CD5L controls a specific subset of inflammatory genes.

Interestingly, recent reports have shown that overexpression of *CD5L* in differentiated THP-1 cells can reduce TNF induction (19) and drive M2 polarization of macrophages (47). However, overexpression of *CD5L* appears to affect the magnitude of TNF induction in response to LPS without affecting its baseline expression. In our *CD5L* deletion cell line, the magnitude of TNF induction is similar to or higher than that of intact THP-1 cells (**Figure 1**), suggesting that the presence of intracellular CD5L was required to define the inflammatory baseline of these macrophages.

We found that the deletion of *CD5L* has remodeled the transcriptome of differentiated THP-1 macrophages (**Figure 3**). A large set of inflammatory mediators was downregulated in the mutant cells, with TNF signaling pathway member genes being most impacted by the *CD5L* deletion. The expression of almost 30% (29 out of 110) of the pathway genes was altered in the deletion cells. This observation suggests that CD5L has a definitive role in the control of inflammatory signaling, and in its absence, there is a global decrease in the basal levels of inflammatory cytokines.

Studies on the lipidome remodeling function of CD5L (6, 13) have prompted us to analyze the extent of changes in lipid content in these cells. Our UPLC–ESI–TOF mass spectrometry analysis of lipids from mutant cells revealed notable changes in lipid content (**Figure 5**).

The limitation of our lipid analysis approach is that we cannot determine the saturation levels of individual side chains for each analyzed lipid. Nevertheless, we could monitor the overall changes in the extent of side chain saturation. While our approach provides an overview of global changes in the levels of side chain saturation, we cannot identify specific acyl groups that are differentially represented in the mutant and control lines. Future analysis using approaches such as tandem mass spectrometry will provide us with the higher resolution needed to identify the mediator of CD5L lipidome remodeling function in human macrophages.

CD5L has been shown to inhibit FASN activity in murine adipocytes, and this has been suggested as a main mechanism by which CD5L remodels the intracellular lipidome in murine Th17 cells (13). Since we observed changes in macrophage lipid content induced by the deletion of *CD5L* (**Figure 5**), we tested whether the same mechanism is used by human macrophages. Surprisingly, we saw no significant differences in FASN activity between mutant and control cells (**Figure 6**). Our result, therefore, suggests that there may be additional mechanisms by which CD5L can control the lipid content of human macrophages.

Our interest in testing the connection between CD5L and members of the ROR nuclear receptor transcription factor family was based on observations made by Wang et al. In a murine model, they discovered that Cd5l acts as a major switch of the inflammatory state of Th17 cells. Murine Cd5l was proposed to control the availability of ligands for RORγt transcription factor (6). Even though CD5L is present at high concentrations in circulation (10), individual Th17 cells maintain a distinct inflammatory state that is determined by the presence or absence of endogenous *Cd5l* expression. Human macrophages do not express RORγt, but produce a closely related RORα member of the ROR family (26). We hypothesized that in macrophages, lipidome remodeling induced by CD5L can be sensed by RORα, similarly to RORγt responding to lipidome changes in Th17 cells. Our earlier work has demonstrated that the deletion of *RORA* has led to an increased basal inflammatory state of THP-1 cells (36). Together with our observation of the opposite inflammatory phenotype of *CD5L* deletion cells, this suggested that lipidome remodeling in the absence of CD5L could be sensed by the RORα nuclear receptor. After deleting *RORA* in *CD5L*-KO cells, we observed that the resulting double knockout and the initial *RORA* deletion cell lines have a similar elevated inflammatory profile (**Figure 2**). However, while RNA-seq analysis revealed a set of divergently expressed genes in *CD5L* and *RORA* deletion cells (**Figure 7**), the testing of ROR response element-containing reporters has shown no difference in their expression between the control cells and CD5Lko. As a result, at this stage, we cannot definitively state that CD5L is directly controlling RORα*-*mediated transcription.

While most inflammation-related studies have focused on macrophages, we were also interested in assessing the effect of CD5L in the precursors of macrophages: monocytes. RNA-seq analysis of undifferentiated *CD5L* deletion cells revealed several differentially expressed genes in mutant cells compared to non-targeting control cells (**Figure 8A**). Interestingly, several of these genes are related to monocyte migration. Among the most significantly upregulated genes was NCAM-1. NCAM-1 is a signaling receptor involved in cell adhesion, has been shown to be strongly upregulated in cardiac myocytes following ischemic insults (48), and has been proposed as a biomarker for coronary artery disease (45). It is also interesting to note that matrix metalloproteinases 2 and 9 (MMP2 and MMP9, respectively) are downregulated (**Figure 8A**). MMP2 and MMP9 are gelatinases with specificity for several extracellular matrix proteins such as various collagen species, elastin, and gelatin (49). MMP2 and MMP9 levels are positively correlated with unstable atherosclerotic plaques, and the proposed contributions to the pathogenesis are both the degradation of the fibrous cap of the plaque and the facilitation of infiltration of immune cells, such as monocytes, into the plaque, exacerbating inflammation (50, 51). The promoter region of MMP9 contains NF-κB binding sites (52), which could explain the observed decreased transcription for this gene in hypo-inflammatory CD5L-KO cells. The expression levels of MMP2 and MMP9 are higher in control cells than in CD5L-KO cells in both differentiated and undifferentiated states (**Figure 8B**); however, the difference is the most pronounced in undifferentiated cells. While differentiation with PMA is inherently pro-inflammatory, there is still a difference between comparable cell states, suggesting that CD5L plays a role in the gene transcription levels. Ishikawa et al. observed decreased expression of MMP2 and MMP9 in *Cd5l*^−/−^ mice following myocardial infarction (53) and proposed that this is due to the depletion of M1 macrophages in the absence of anti-apoptotic CD5L function. In contrast, in our experiments, we observed a baseline reduction in the levels of these gene transcripts.

Several papers have linked CD5L to atherosclerosis development (54). While reduced atherosclerotic lesions and foam cell numbers have been observed in *Cd5l*^−/−^/*Ldlr*^−/−^ mice, the main proposed proatherogenic mechanism for CD5L is that of apoptosis inhibition and facilitation of oxidized LDL uptake (18). Our data suggest that CD5L may also control cell migration through MMP2, MMP9, and NCAM-1 regulation, and this could also be a factor in atherogenesis through reduced monocyte recruitment to the plaque.

**Figure 9:**
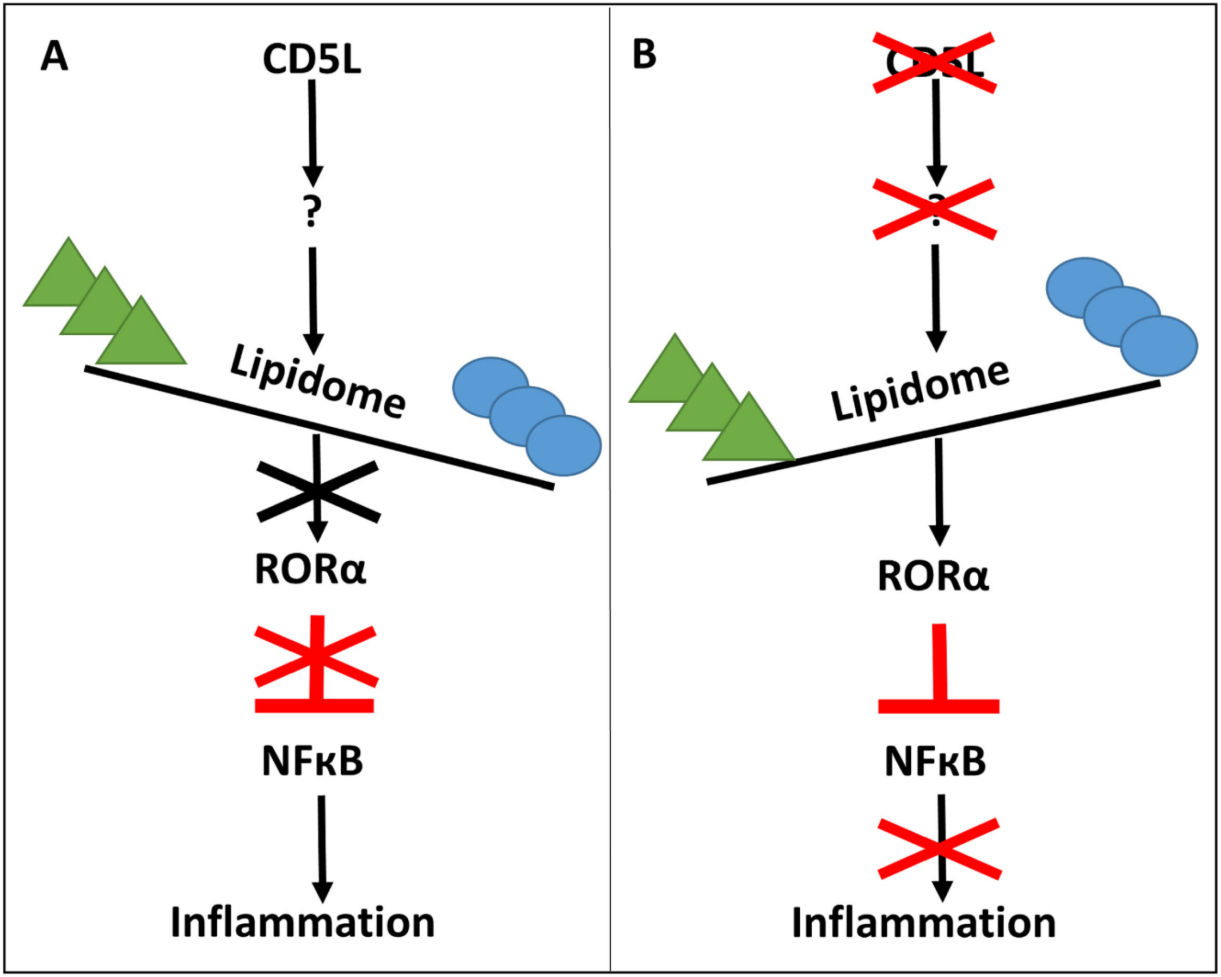
Model for the role of CD5L in mediating inflammatory state of human macrophages. **(A)** CD5L induces changes in macrophages’ lipid content, reducing the availability of RORα ligands and subsequent RORα inactivation. Thus, no inhibitory effect of RORα is applied on inflammatory signaling. **(B)** In the absence of CD5L, there are sufficient levels of RORα ligands driving its activation and downregulation of inflammatory signaling.

Our analysis of *CD5L* and *RORA* deletion cells, along with earlier evidence suggesting negative regulation of NF-κB signaling by RORα (35), suggested a model of how endogenous CD5L regulates metabolic and inflammatory states in macrophages (**Figure 9**). In our model, the presence or absence of endogenous *CD5L* expression determines the lipid content of the cells. This, in turn, results in changes in the availability of endogenous RORα ligands. In the absence of RORα activity, there is reduced inhibition of NF-κB inflammatory signaling, driving a hyper-inflammatory state of cells (**Figure 9A**). Conversely, in the absence of *CD5L* expression, RORα ligands are available. Active RORα inhibits inflammation to downregulate pro-inflammatory cytokines (**Figure 9B**). The majority of our data support this model in which CD5L determines the macrophage inflammatory profile by modulating RORα activity. Nevertheless, we were unable to show CD5L-dependent changes in the transcriptional activity of RORE reporters, and therefore, we still need to generate formal proof of our hypothesis.

While comparing changes in gene expression in monocytic and macrophage-like THP-1 cells deleted for *CD5L*, we noticed a strong induction of *CD52* transcripts in both datasets. This induction is notable because it has been recently discovered that the soluble form of CD52 strongly inhibits TLR-mediated activation of NF-κB as well as triggers apoptosis (46). Such effects of CD52 induction are consistent with our observed suppression of inflammation in CD5L knockouts as well as previously documented involvement of CD5L in the inhibition of apoptosis (14). It is also interesting that a known RORα ligand, all-*trans* retinoic acid (ATRA), has been shown to induce CD52 (55), while in the absence of RORα, CD52 induction is dampened (56). Furthermore, the RNA-seq dataset generated in our earlier study of the *RORAko* in THP-1 cells also showed that the absence of RORα leads to decreased expression of *CD52* (36). All these observations identify CD52 as a strong novel candidate for a mediator of CD5L-dependent control of macrophage inflammatory state.

In our study, we analyzed CD5L function in the THP-1 monocyte-like cell line primarily because it is a widely used and well-characterized model system amenable to genetic manipulation. However, because this is an immortalized line, it has several features that are different from primary human monocytes (41). Therefore, results obtained in this model system should be independently validated. BLaER1 cell line can be transdifferentiated into human monocytes, which recapitulate many aspects of primary cells (57). It is becoming widely accepted as a functional model to study monocyte function and is therefore a great candidate for future analysis of the role of CD5L in the control of inflammatory state of human immune cells.

CD5L is a secreted molecule abundant in circulation (10–12). Several studies using recombinant CD5L have demonstrated that exogenously delivered recombinant CD5L is capable of modulating the inflammatory properties of immune cells (58). The results of our study can therefore reflect both the endogenous and extracellular functions of CD5L. Considering that all our experiments were carried out in the presence of 10% FBS, which normally contains bovine CD5L, we speculate that the differences in the levels of secreted CD5L within the timeframe of our experiments should be buffered by the bovine protein in serum. Nevertheless, this question needs to be addressed in the future using serum-free cell culture with and without recombinant CD5L supplementation.

Our data provide further support for the key role of CD5L in controlling the inflammatory state of human macrophages and their lipid content. While we were not able to show the direct effect of CD5L absence on the regulation of gene expression from promoters containing ROR response elements, our results nevertheless suggest a novel connection between inflammatory and metabolic signaling in human macrophages that is dependent on CD5L and RORα. Our data, therefore, provides yet another piece of evidence of the tight multi-level integration of metabolism and inflammation in immune cells.

## Data Availability

The datasets presented in this study can be found in online repositories. The names of the repository/repositories and accession number(s) can be found below: https://www.ebi.ac.uk/arrayexpress/, E-MTAB-7295.
