# Supplementary material for: Endogenous CD5L controls the metabolic and inflammatory state of human macrophages
Source: Front Immunol. 2026 Jan 22;16:1677948. doi: 10.3389/fimmu.2025.1677948 (PMC12872570; doi:10.3389/fimmu.2025.1677948)
Supplement: Supplementary file 1 [file SupplementaryFile1.docx]

**Figure S1**

1. **CD5L mutant clones:**

Sequence of exon 3 for Clone 1 and 2:

CCAGTGGGGCACCGTGTGTGATGACGGCTGGGACATTAAGGACGTGGCTGTGTTGTGCCGGGAGCTGGGCTGTGGAGCTGCCAGCGGAACCCCTAGTGGTATTTTGTATGAGCCACCAGCAGAAAAAGAGCAAAAGGTCCTCATCCAATCAGTCAGTTGCACAGGAACAGAAGATACATTGGCTCAGTGTGAGCAAGAAGAAGTTTATGATTGTTC

Sequence of exon 3 for clone 3:

CCAGTGGGGCACCGTGTGTGATGACGGCTGGGACATTAAGGACGTGGCTGTGTTGTGCCGGGAGCTGGGCTGTGGAGCTGCCAGCGGAACCCCTAGTGGTATTTTGTATGAGCCACCAGCAGAAAAAGAGCAAAAGGTCCTCATCCAATCAGTCAGTTGCACAGGAACAGAAGATACATTGGCTCAGTGTGAGCAAGAAGAAGTTTATGATTGTTC

Sequence of exon 4 for clone 1, 2 and 3:

GAAGCACCAGAACCAGTGGTATACCGTGTGCCAGACAGGCTGGAGCCTCCGGGCCGCAAAGGTGGTGTGCCGGCAGCTGGGATGTGGGAGGGCTGTACTGACTCAAAAACGCTGCAACAAGCATGCCTATGGCCGAAAACCCATCTGGCTGAGCCAGATGTCATGCTCAGGACGAGAAGCAACCCTTCAGGATTGCCCTTCTGGGCCTTGGGGGAA

1. **RORα mutant clone:**

Sequence of exon 4:

GCCAACACTGTCGATTACAGAAATGCCTTGCCGTAGGGATGTCTCGAGATGGTGAGCTCTCACAGCTTGAGT

: Frameshift leads to premature stop codon 5 amino acids downstream.

Sequence of exon 5:

GAGAGGCTGAGCCGCTGACGCCCACCTACAACATCTCGGCCAACGGGCTGACGGAACTTCACGACGACCTCA

1. **CD5L/ RORα mutant clone:**

Sequence of exon 4: (same as exon 4 in CD5L KO clones)

GAAGCACCAGAACCAGTGGTATACCGTGTGCCAGACAGGCTGGAGCCTCCGGGCCGCAAAGGTGGTGTGCCGGCAGCTGGGATGTGGGAGGGCTGTACTGACTCAAAAACGCTGCAACAAGCATGCCTATGGCCGAAAACCCATCTGGCTGAGCCAGATGTCATGCTCAGGACGAGAAGCAACCCTTCAGGATTGCCCTTCTGGGCCTTGGGGGAA

**S1 Fig.** **Sequencing of the CRISPR/Cas9 targeted regions in *CD5L*, *RORA* and *CD5L/RORA* mutant cells**. Nucleotides shaded in grey are deleted in respective clones. Blue boxes outline guide RNA sequences.

**Figure S2**


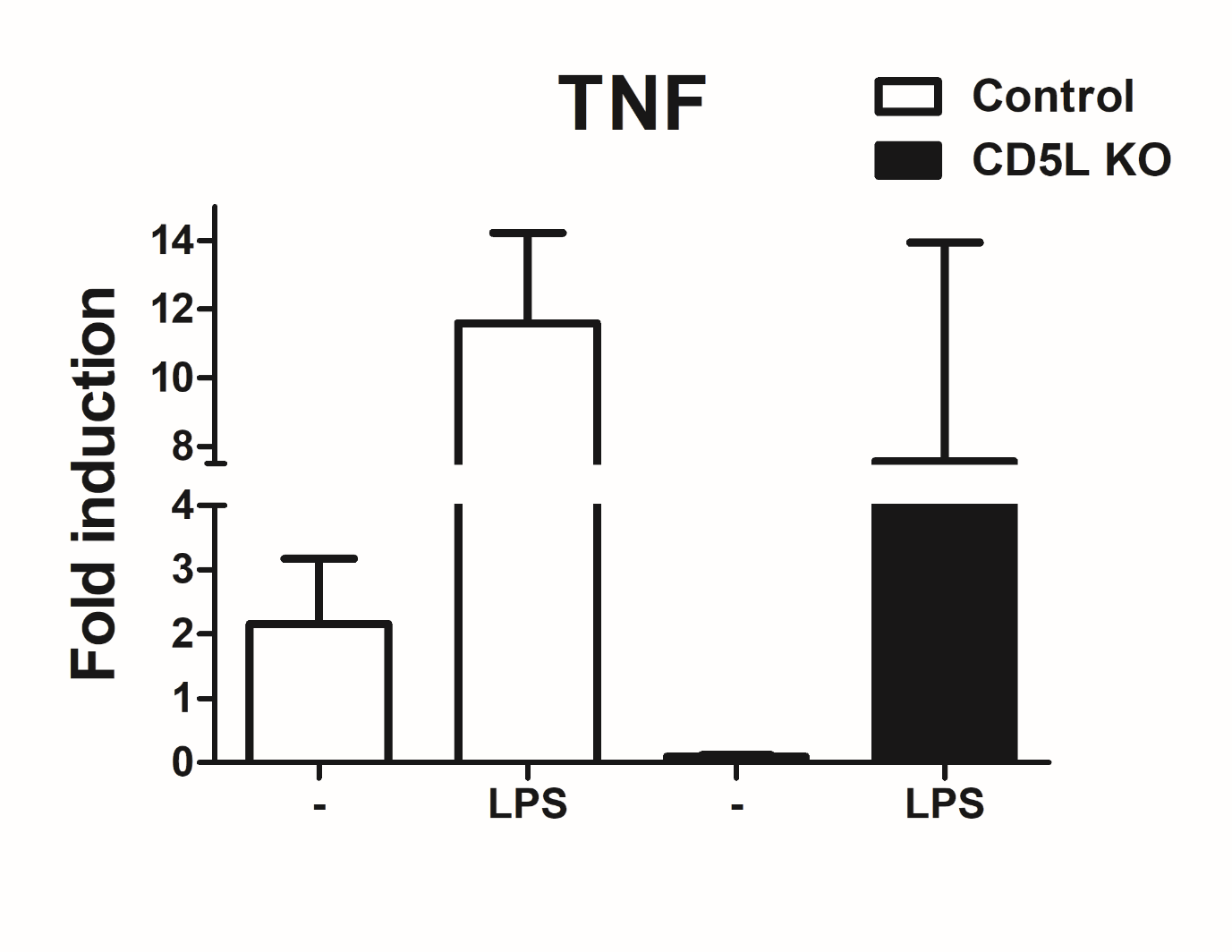
**S2 Fig.** ***TNF* expression levels in undifferentiated *CD5L*-deletion cells**. Protein levels of TNF were nearly 25-fold lower in supernatants of unstimulated mutant cells.


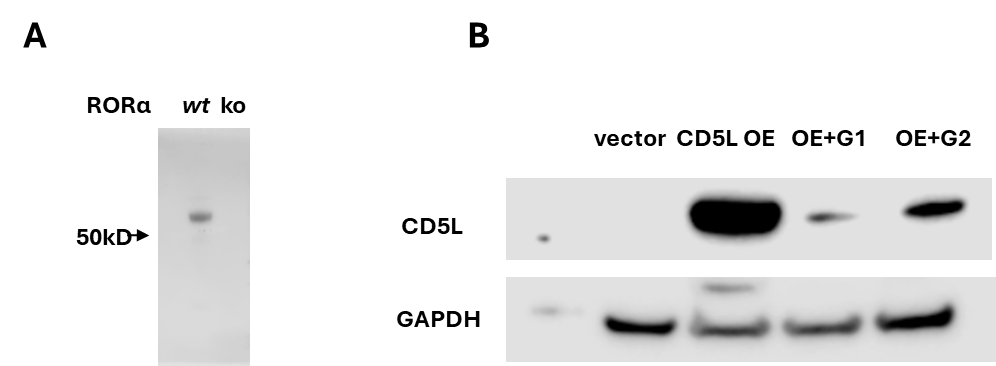


**S3 Fig. Western blot of knockout cell lines**

A) Cell line with RORA gene targeted by CRISPR Cas9 guides show no detectable protein B) Both G1 and G2 gene specific guides (Table 1) efficiently ablate CD5L coding sequence as illustrated by nearly complete disappearance of overexpressed human CD5L (CD5LOE)

**Table S1**

| **Gene** | **ENSEMBL ID** | **Description** |
| --- | --- | --- |
| AXL | ENSG00000167601 | AXL receptor tyrosine kinase |
| CD52 | ENSG00000169442 | CD52 molecule |
| DACH1 | ENSG00000276644 | dachshund family transcription factor 1 |
| FGL2 | ENSG00000127951 | fibrinogen like 2 |
| HMX3 | ENSG00000188620 | H6 family homeobox 3 |
| HSH2D | ENSG00000196684 | hematopoietic SH2 domain containing |
| ISYNA1 | ENSG00000105655 | inositol-3-phosphate synthase 1 |
| MNX1 | ENSG00000130675 | motor neuron and pancreas homeobox 1 |
| MSRB3 | ENSG00000174099 | methionine sulfoxide reductase B3 |
| NCAM1 | ENSG00000149294 | neural cell adhesion molecule 1 |
| NPTX1 | ENSG00000171246 | neuronal pentraxin 1 |
| PENK | ENSG00000181195 | proenkephalin |
| PTK7 | ENSG00000112655 | protein tyrosine kinase 7 (inactive) |
| SIGLEC6 | ENSG00000105492 | sialic acid binding Ig like lectin 6 |
| SRGN | ENSG00000122862 | serglycin |
| UNCX | ENSG00000164853 | UNC homeobox |

**S1 Table.**  Genes significantly upregulated more than 2 fold in differentiated and undifferentiated CD5Lko cells

**Table S2**

| Guide | Sequence |
| --- | --- |
| CD5L guide 1 (KO1) | CCTCCACCGCTGTGAAGGGC |
| CD5L guide 2 (KO2) | CGGCTGGGACATTAAGGACG |
| Non-targeting | TTTGTAATCGTCGATACCC |

**S2 Table.** Guides used for deletion of CD5L in undifferentiated THP-1 cells
